# Insulin Resistance Is Associated with Intraocular Pressure Elevation in a Non-Obese Korean Population

**DOI:** 10.1371/journal.pone.0112929

**Published:** 2015-01-05

**Authors:** Yoon Hong Chun, Kyungdo Han, Shin Hae Park, Kyung-Min Park, Hyeon Woo Yim, Won-Chul Lee, Yong Gyu Park, Yong-Moon Park

**Affiliations:** 1 Department of Pediatrics, Incheon St. Mary's Hospital, College of Medicine, The Catholic University of Korea, Seoul, Republic of Korea; 2 Department of Biostatistics, The Catholic University of Korea, Seoul, Republic of Korea; 3 Department of Ophthalmology and Visual Science, Seoul St. Mary's Hospital, College of Medicine, The Catholic University of Korea, Seoul, Republic of Korea; 4 School of Medicine, The Catholic University of Korea, Seoul, Republic of Korea; 5 Department of Preventive Medicine, College of Medicine, The Catholic University of Korea, Seoul, Republic of Korea; 6 Department of Epidemiology and Biostatistics, Arnold School of Public Health, University of South Carolina, Columbia, South Carolina, United States of America; Weill Cornell Medical College Qatar, Qatar

## Abstract

Based on reports of an association between elevated intraocular pressure (IOP) and metabolic syndrome (MetS), and the major role of insulin resistance (IR) in MetS pathogenesis, a positive association between IOP and IR has been hypothesized. Although Asian populations tend to have lower body mass indices (BMIs) than Western populations, they tend to have a higher risk of developing MetS. This study examined the hypothesis that the association between IOP and IR differs by obesity status in an Asian population, by examining a nationally representative sample of South Korean adults. Data collected from 4,621 South Korean adults regarding demographic, lifestyle, and laboratory parameters by the 2010 Korea National Health and Nutrition Examination Survey were subjected to linear regression analysis to evaluate the relationship between IOP and metabolic profiles. After adjusting for confounding factors, the data were subjected to multiple linear regression analysis to examine the association between IR, as measured by the homeostasis model assessment of insulin resistance (HOMA-IR), and IOP. Obesity was defined as BMI≥27.5 kg/m^2^, and the subjects were divided into obese vs. non-obese groups for investigation of the association between IR and IOP according to obesity status. IOP was found to correlate with fasting blood sugar, total cholesterol, insulin, and HOMA-IR values in non-obese men; and with BMI, waist circumference, triglycerides, total cholesterol, HOMA-IR, and low-density lipoprotein cholesterol values in non-obese women, whereas no association between IOP and IR was found in obese men or women. IOP was significantly associated with IR in non-obese men and women after adjusting for age, and in non-obese men after adjusting for age, BMI, and lifestyle and demographic factors. These findings indicate that a positive and independent relationship exists between IOP and IR in non-obese individuals only, suggesting that other factors likely contribute to IOP elevation in obese individuals.

## Introduction

Intraocular pressure (IOP) is determined by the balance between aqueous humor secretion and outflow, and is closely associated with the development of glaucomatous optic nerve damage [1], [2]. As it can be lowered by medications or surgery, IOP is the only known modifiable risk factor for glaucomatous optic nerve damage [3]. However, the pathophysiology underlying IOP elevation is still not fully understood, and identifying the parameters that can be modified to lower IOP is therefore clinically important.

Many cross-sectional and longitudinal epidemiologic studies have reported an association between elevated IOP and metabolic syndrome (MetS) [4]–[6], a condition characterized byinsulin resistance (IR), hyperinsulinemia, abdominal adiposity, dyslipidemia (elevated triglycerides and low high-density lipoprotein [HDL] cholesterol), elevated fasting glucose, cardiovascular disease, hypertension, certain cancers, and increased mortality [7],[8]. Based on observations that IR plays a major role in the pathogenesis of MetS, a positive association between IOP and IR has also been hypothesized. In support of this hypothesis, a previous hospital-based study investigating the link between IOP and IR found a positive association between IR indices and IOP, even after adjusting for other risk factors [9]. However, this study had several potential limitations, including selection bias due to the relatively small sample and its single-center design.

Evidence from several prospective studies in Asia suggests that although individuals of Asian descent generally have a lower body mass index (BMI) than individuals of European descent, they are at higher risk of developing MetS [10]. In accordance with this evidence, the onset of obesity-related complications has been observed in Asian populations at much lower BMIs [11],[12]. Based on these findings, we hypothesized that the association between IOP and IR might differ among individuals, especially among individuals of Asian descent, according to obesity status. To test this hypothesis, we analyzed the associations between IOP and IR in obese and non-obese individuals in a nationally representative sample of the South Korean population, while adjusting for other risk factors. Our analysis yielded several findings with important clinical and research implications.

## Materials and Methods

### Study subjects

This study examined data collected by the 2010 Korea National Health and Nutrition Examination Survey (KNHANES), a cross-sectional survey conducted by the Korea Center for Disease Control and Prevention and approved by its institutional review board. The 2010 KNHANES used a multistage, stratified, probability-clustered sampling method and a weighting scheme that allowed for the estimation of health statistics representative of the civilian, non-institutionalized South Korean population. Additional details regarding the study design and methods have been provided elsewhere [13],[14]. All participants in the KNHANES provided written informed consent prior to study initiation.

The subjects of the current study were selected from 8,477 individuals, representing 77.5% of the target population of 10,938 individuals who had participated in the 2010 KNHANES. Among these, 6,283 subjects who were 19 years and older and who had undergone thorough ophthalmic examinations were selected. Those with a history of medical treatment for diabetes (n = 926), high fasting blood sugar levels (n = 123), renal failure (n = 2), liver disease (n = 29), pregnancy (n = 17), malignant tumor (n = 57), glaucoma (n = 13) and/or a history of ophthalmic surgery (n = 406), or for whom data were missing (n = 89), were excluded from further analysis, yielding a final sample of 4,621 subjects.

### Data collection

The 2010 KNHANES consisted of three main components, namely a health interview; health examination, which included ophthalmologic interview questions and examinations; and a nutrition survey. All KNHANES interviews and examinations were performed by trained staff according to standardized procedures. Questionnaires were administered to the participants to collect data regarding demographic characteristics, smoking status, alcohol consumption, daily exercise level, residential status (urban or rural), educational level, family income, occupation, family history of glaucoma, daily sun exposure, and daily nutritional intake. Additional surveys were administered to the female participants to collect data regarding menopausal status and use of hormonal replacement therapy. Based on self-reported smoking behavior, subjects were categorized as either current smokers or nonsmokers. Based on self-reported sun exposure time, subjects were classified as experiencing less than 2 hours, 2–5 hours, and more than 5 hours of sun exposure per day. Using self-reported alcohol consumption data, the average frequency and quantity of alcohol consumed was converted into pre-alcohol grams consumed per day, and the subjects were classified as either nondrinkers/moderate drinkers (0–30.0 g alcohol/day) or heavy drinkers (>30.0 g alcohol/day). Based on their responses to a modified version of the International Physical Activity Questionnaire, the subjects were classified as either regular exercisers or not regular exercisers. Based on specification whether they lived in a city of any size or did not live in a city, the subjects were classified as living in an urban residence or in a rural residence, respectively. Based on self-reported educational level, subjects were classified as having either a low (less than high school or ninth-grade education) or a high (ninth-grade education or above) educational level. Using self-reported per capita household income data, household income was inflation-adjusted and measured as quartiles to classify subjects into the highest, middle-high, middle-low, and lowest quartiles.

All anthropometric measurements were performed by a specially-trained examiner. Waist circumference (WC) was measured at the mid-point between the lower margin of the last palpable rib and the top of the iliac crest at the end of a normal expiration with the arms relaxed at the sides. BMI was calculated as weight in kilograms divided by the square height in meters. Laboratory testing during the survey period was performed by collecting blood samples from each participant in the morning after at least an 8-h fast and then processed, immediately refrigerated, and transported in a cold storage to the Central Testing Institute in Seoul, Korea. Analysis of all blood samples was performed within 24 h after transportation. Measurement of concentrations of fasting blood sugar (FBS), HDL cholesterol, low-density lipoprotein (LDL) cholesterol, total cholesterol (TC), and triglycerides (TG) were performed using a Hitachi automatic analyzer 7600 (Tokyo, Japan). Immunoradiometric assays using a 70 WIZARD gamma-counter (PerkinElmer, Finland) were used for the measurement of insulin. The homeostasis model assessment of insulin resistance (HOMA-IR) value was calculated using the following formula: HOMA-IR = FBS (mg/dL)×fasting insulin (µIU/mL)/405 [13]. Individuals in the highest quartile of HOMA-IR were defined as insulin resistant, and those in the lowest quartile as insulin sensitive. The quartile HOMA-IR values were calculated by sex, as described previously [15],[16]. Obesity was defined as a body mass index of 27.5 kg/m^2^, the cutoff reportedly associated with increased mortality rates in Asians, including Koreans [17],[18]. Ophthalmologic examinations were performed in a mobile examination unit by a trained ophthalmologist or ophthalmology resident. IOP was measured once per eye by study ophthalmologists during slit-lamp examination with a Goldmann applanation tonometer (Haag-Streit, Inc., Bern, Switzerland).

### Statistical analysis

SAS version 9.2 software and survey procedures (SAS Institute, Inc., Cary, NC, USA) were used to perform all statistical analyses. Sampling weights were used to reflect the complex sampling design of the KNHANES and to acquire nationally representative estimates. Domain analysis was performed to preserve the complex sampling design when analyzing entire samples for estimating the variance of subpopulations in the subgroup analysis. The data are presented as mean ± standard error (SE) or proportion (SE) for continuous and categorical variables, respectively. The characteristics of the study subjects were compared by sex. Variables with skewed distributions, such as the HOMA-IR and TG values, were analyzed after logarithmic transformation. Mean IOP was compared according to the HOMA-IR quartiles in the non-obese and obese groups by ANOVA. Simple linear regression analyses were performed to evaluate the linear relationship between IOP and metabolic profiles. Multiple linear regression analyses were performed to examine the association between IR and IOP after adjusting for age, BMI, smoking status, alcohol consumption, exercise level, education, income, family history of glaucoma, sun exposure, daily calorie intake, and percentage of dietary fat in the daily nutritional intake. Menopausal status and hormonal replacement therapy were used as covariates for the female subjects. Subgroup analyses of obese and non-obese subjects were conducted based on the hypothesis that the association between IR and IOP may differ by obesity status. Sensitivity analysis was performed to identify the existence of any differential association between IR and IOP according to menopausal status in women. For all analyses, a *p*-value <0.05 was considered statistically significant.

## Results

The demographics of the study subjects are summarized in Table 1. In accordance with previous reports that IR-related metabolic characteristics differ by sex [19],[20], the interaction term between IR and sex was found to be significant (p = 0.0164). Statistically significant intrasex differences were moreover found regarding laboratory, demographic, and lifestyle-related characteristics, including for age, BMI, WC, IOP, FBS level, TG level, HOMA-IR values, HDL level, smoking status, alcohol consumption, exercise level, educational level, family income, employment status, sun exposure, and the percentage of daily fat intake (for female subjects only).

**Table 1:** General characteristics of the study subjects.

	Male			Female		
	Non-obese	Obese	P-value	Non-obese	Obese	P-value
	(n = 1272)	(n = 719)		(n = 1949)	(n = 681)	
**Age (years)**	42.2±0.6	42.1±0.7	<.0001	42.1±0.5	48.5±0.7	<.0001
**BMI (Kg/m^2^)**	22.1±0.1	27.4±0.1	<.0001	21.5±0.1	27.7±0.1	<.0001
**WC (cm)**	79.2±0.2	91.8±0.4	<.0001	73.3±0.3	87.8±0.4	<.0001
**IOP (mmHg)**	14.1±0.1	14.5±0.2	<.0001	13.7±0.1	14.1±0.2	<.0001
**FBS (mg/dl)**	91.7±0.4	96±0.4	<.0001	89.5±0.2	94.7±0.5	<.0001
**TG (mg/dl)**	108.2 (103.6,113)	156.2 (147.9,164.9)	<.0001	81.3 (79.1, 83.7)	115.2 (109.4, 121.2)	<.0001
**TC (mg/dl)**	182.2±1.2	197.2±1.6	<.0001	181.5±1	198.1±1.8	<.0001
**Insulin (µIU/mL)**	8.6 (8.4,8.8)	11.5 (11.1, 11.9)	<.0001	9 (8.8, 9.2)	11.4 (11, 11.8)	<.0001
**HOMA-IR**	1.9 (1.9,2)	2.7 (2.6, 2.8)	<.0001	2 (1.9, 2)	2.6 (2.5, 2.8)	<.0001
**HDL (mg/dl)**	51.8±0.5	46.9±0.5	<.0001	57.8±0.4	51.9±0.5	<.0001
**LDL(mg/dl)**	109.1±1.1	119.4±1.6	<.0001	110.2±0.8	124.3±1.5	<.0001
**Current smokers (%)**	44 (1.5)	41.4 (2.4)	0.3393	5 (0.6)	6.1 (1.4)	0.4334
**Heavy drinkers (%)**	16.2 (1.1)	20.8 (1.8)	0.0262	1.6 (0.4)	3.3 (0.9)	0.0320
**Regular exercisers (%)**	25.4 (1.5)	25.5 (2.1)	0.9735	18.6 (1.2)	26 (2.1)	0.0002
**Rural residence (%)**	22.1 (3.5)	19.6 (3.3)	0.3238	19.5 (3)	26 (4)	0.0047
**Low education (%)**	21.3 (1.6)	16 (1.6)	0.0100	26.3 (1.7)	47.6 (2.5)	<.0001
**Lowest income (%)**	13.3 (1.4)	11 (1.4)	0.1906	14.9 (1.1)	22.9 (2.3)	0.0002
**Employed (%)**	80.1 (1.5)	84.5 (2)	0.0824	54.9 (1.6)	53.9 (2.2)	0.7173
**Glaucoma FHx (%)**	2.5 (0.5)	2.1 (0.7)	0.6504	2.5 (0.4)	1.4 (0.5)	0.1624
**Sun exposure**			0.6761			0.8443
**<2 h**	55.3 (2.1)	54.4 (2.2)		70.9 (1.5)	69.6 (2.5)	
**2 to 5 h**	28.5 (1.7)	27.6 (2)		20.5 (1.2)	21.2 (2)	
**≥5 h**	16.1 (1.6)	18 (1.8)		8.6 (1.2)	9.2 (1.5)	
**Post menopause (%)**				26.5 (1.5)	42.5 (2.7)	<.0001

Data are presented as mean ± standard deviation or n (%).

Abbreviations: BMI = Body Mass Index, WC = Waist circumference, IOP = Intraocular pressure, FBS = Fasting Blood Sugar, TG = Triglycerides, TC = Total Cholesterol, HOMA-IR = Homeostasis Model Assessment of Insulin Resistance, HDL = High Density Lipoprotein Cholesterol, LDL = Low Density Lipoprotein Cholesterol, FHx = Family History.

The correlations between IOP and BMI, dyslipidemia components, and HOMA-IR values according to obesity status (obese vs. non-obese) are shown in Table 2. In non-obese men, IOP was found to correlate with FBS level (p = 0.014), TC level (p = 0.004), insulin level (p = 0.031), and log of HOMA-IR value (p = 0.091). In non-obese women, IOP was found to correlate with BMI (p = 0.017), WC (p = 0.001), the log of TG level (p = 0.043), the TC level (0.002), the HOMA-IR value (p = 0.034), and the LDL level (p = 0.007). However, no correlations were found between IOP and any of the variables examined in obese men and women.

**Table 2:** Association between IOP and metabolic profile components by sex and obesity status.

	Male				Female			
	Non obese		Obese		Non obese		Obese	
	r*	P-value§	r*	P-value§	r*	P-value§	r*	P-value§
**Age**	0.05117	0.1320	−0.04081	0.3726	0.05294	0.0615	−0.10913	0.0376
**BMI**	0.04562	0.1563	0.05849	0.2437	0.07316	0.0170	0.03797	0.4403
**WC**	−0.01450	0.4253	0.03598	0.4492	0.11830	0.0010	−0.00446	0.9199
**FBS**	0.09819	0.0135	0.06424	0.1214	0.04040	0.1235	0.00631	0.9082
**Log TG**	0.06285	0.0803	0.05340	0.2719	0.05073	0.0426	0.01565	0.7074
**TC**	0.08879	0.0042	0.07498	0.0643	0.08702	0.0022	−0.03566	0.5219
**Log Insulin**	0.07505	0.0305	0.00764	0.8531	0.05298	0.0645	−0.02564	0.6052
**Log HOMA-IR**	0.09122	0.0078	0.02143	0.6026	0.05783	0.0344	−0.02318	0.6494
**HDL**	0.01837	0.5528	0.05214	0.2461	0.01490	0.6116	−0.0258	0.5371
**LDL**	0.05769	0.0807	0.02490	0.5339	0.07761	0.0065	−0.03447	0.5210

* Regression coefficient.

§ p, value calculated by simple linear regression analysis.

Abbreviations: BMI = Body Mass Index, WC = Waist circumference, IOP = Intraocular pressure, FBS = Fasting Blood Sugar, TG = Triglycerides, TC = Total Cholesterol, HOMA-IR = Homeostasis Model Assessment of Insulin Resistance, HDL = High Density Lipoprotein Cholesterol, LDL = Low Density Lipoprotein Cholesterol.

To evaluate the relationship between IOP and IR, multiple linear regression analyses were performed. As shown in Table 3, IR was found to be independently and positively associated with IOP in non-obese men (p = 0.008) and non-obese women (p = 0.034) after adjusting for age. After adjusting for age, BMI, alcohol consumption, smoking status, exercise level, education level, income, family history of glaucoma, sun exposure, and daily calorie intake; as well as the percentage of daily fat intake, menopausal status, and hormonal replacement therapy (female subjects only), IR remained independently and positively related to IOP in non-obese men (p = 0.048) and non-obese women (p = 0.045). In contrast, no associations were found between IOP and IR in either obese men or obese women.

**Table 3:** Multiple linear regression analysis of the associations between mean intraocular pressure and insulin resistance by sex and obesity status.

	Non obese				Obese		
	Beta*	SE	P-value		Beta*	SE	P-value
				**Male**			
MODEL1§	0.680466	0.252759	0.0078		0.142091	0.272408	0.6026
MODEL2¥	0.659045	0.260247	0.0123		−0.028270	0.312774	0.9281
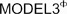	0.527542	0.265365	0.0484		0.343015	0.344363	0.3206
				**Female**			
MODEL1§	0.424281	0.198966	0.0344		−0.141510	0.310742	0.6494
MODEL2¥	0.339676	0.200434	0.0920		−0.206070	0.327547	0.5301
MODEL4†	0.435297	0.215540	0.0450		−0.144795	0.406476	0.7221

* Standardized coefficient.

§ Model 1: adjusted for age.

¥ Model 2: adjusted for age, body mass index, smoking status, alcohol consumption, exercise level, educational level, and family income.

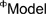
 3: adjusted for age, body mass index, smoking status, alcohol consumption, exercise level, educational level, family income, family history of glaucoma, and sun exposure, daily calorie intake, and daily fat intake.

† Model 4: adjusted for age, body mass index, smoking status, alcohol consumption, exercise level, educational level, family income, family history of glaucoma, sun exposure, daily calorie intake, daily fat intake, menopause, and hormonal replacement therapy.

Abbreviations: SE = standard error.

**Figure 1:**
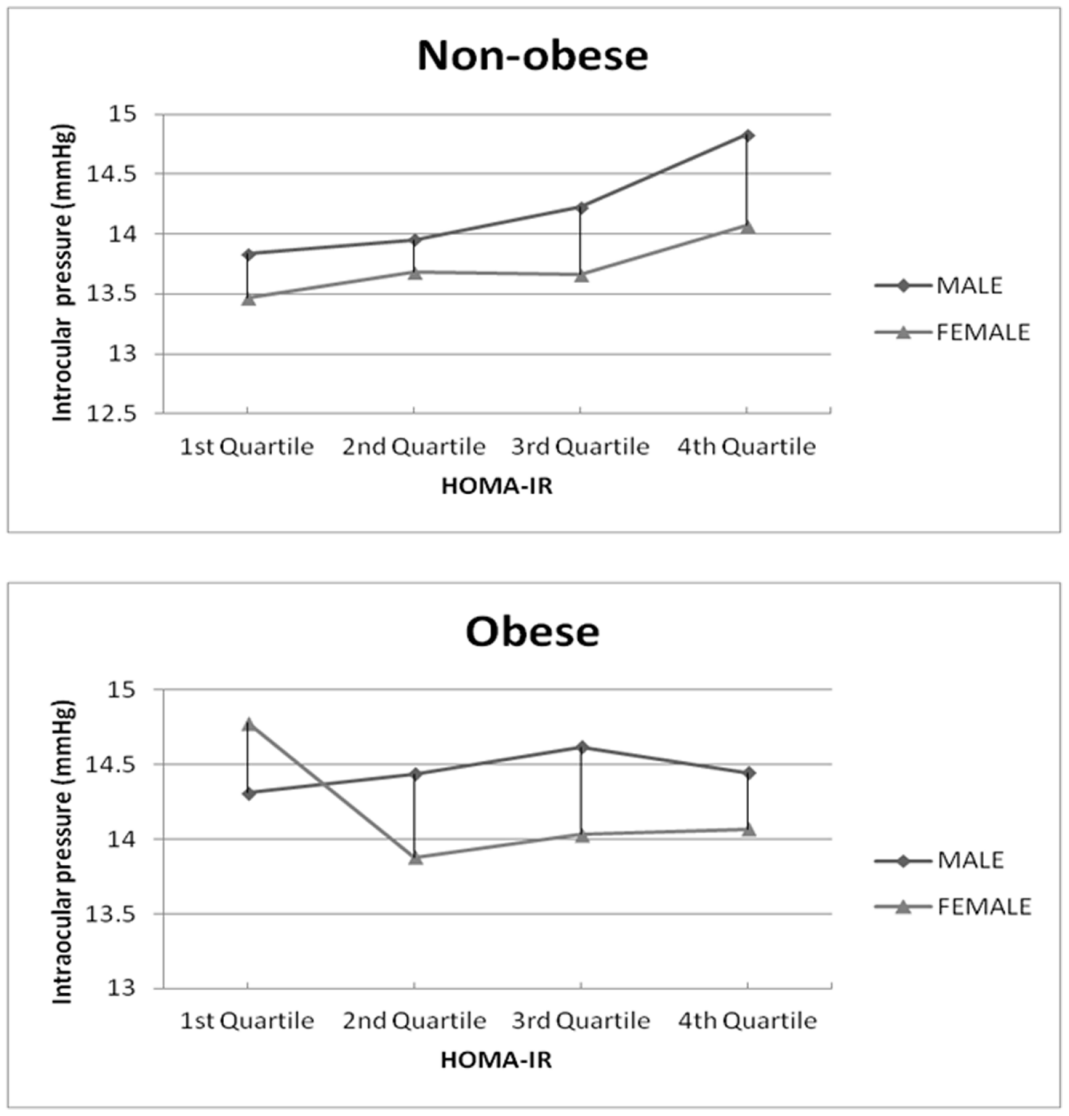
Changes in intraocular pressure according to insulin resistance quartiles in non-obese and obese men and women. The linear graph shows a gradual increase in the mean intraocular pressure with the higher quartile of insulin resistance in non-obese men and women (p for trend = 0.0006 for men; 0.0096 for women), but not in obese men and women (p for trend = 0.8121 for men; 0.3596 for women).

As shown in Fig. 1, gradual increases in the mean IOP according to the HOMA-IR quartiles, from the lowest to the highest quartile, were observed in non-obese men (p for trend = 0.010) and non-obese women (p for trend = 0.007), but not in obese men (p for trend = 0.725) or obese women (p for trend = 0.609).

## Discussion

In this cross-sectional study, a positive and independent relationship was observed between IOP and IR in non-obese subjects but not in obese subjects, in whom no association was observed. In non-obese men, the positive association between IOP and IR was robust, and this association was observed even after adjusting for age, BMI, smoking status, alcohol consumption, exercise level, family income, family history of glaucoma, and sun exposure.

As a positive association between IOP and MetS has been observed in both Western and Asian populations [4]–[7],[17],[18],[20],[21], and as MetS is known to be strongly associated with IR, IR has been hypothesized to be a contributing factor to ocular hypertension [9]. Although the mechanism by which IR affects IOP remains unclear, one hypothesis is that IR stimulates ocular sympathetic activity [22], leading to hyperactivation and potentially to IOP elevation. Previous studies have demonstrated that moderate stimulation of the cervical sympathetic nerves can increase the IOP in rabbits [23] and that it may transiently increase the IOP before decreasing the IOP in cats [24]. The mechanism underlying the IOP increases observed in these studies is hypothesized to be contraction of the extraocular muscles [25]. Unfortunately, the IOP responses to electrical stimulation of various frequencies have not yet been evaluated in humans. However, a previous study found a statistically significant association between BMI and IOP in models excluding IR indices as covariates, but not in models including IR indices, suggesting that the association between BMI and IOP might reflect the existence of a true association between IR and IOP [9].

Surprisingly, no association was found between IR and IOP in the obese subjects examined in the current study. This finding suggests that IR and obesity may be independent contributors to IOP elevation; or, at the very least, that IR may play only a minor, or no, role in the positive association between obesity and IOP. A previous study identified obesity as an independent risk factor for IOP elevation and proposed the existence of several mechanisms underlying the positive relationship between IOP and obesity [26]. One proposed mechanism is that the accumulation of excessive intraorbital adipose tissue in obesity increases IOP by increasing blood viscosity and episcleral venous pressure; and, consequently, decreasing the outflow of aqueous humor [27],[28]. Abdominal obesity is also known to be a major contributor to diabetes, hypertension, and MetS, in which IR is believed to play a large part in the pathophysiology [8]. Moreover, many studies of the association between MetS and IOP have observed a higher mean IOP in subjects with a greater number of MetS components [8],[9].

Despite these findings, it remains unclear whether the association between obesity and IOP elevation results from the existence of a mechanical or a biochemical mechanism or from pathogenesis. Regarding the former, certain mechanical factors, such as the restricted orbital and thoracic space in obesity, have been proposed to play a role in IOP elevation.

An alternative explanation is that BMI does not necessarily directly correlate with IR. Recent evidence suggests an association of IR with abdominal obesity [29]; and, as centrally located visceral fat affects IR more strongly than centrally located subcutaneous fat [30], abdominal obesity, in particular, places individuals at higher risk for developing IR. Moreover, the association between obesity and IR has been reported to be largely the result of changes in the function of adipose tissue, specifically increased release of free fatty acids and abnormalities in adipokine secretion [31]. These findings, along with the differences in the association between IOP and IR according to obesity status observed in the present study, suggest that obesity might be associated with IOP elevation primarily because of the influence of anatomic or physiologic factors secondary to obesity.

Other hypotheses include that the decrease in aqueous outflow and the increase in intraorbital adipose tissue, episcleral pressure, and blood viscosity due to increased hematocrit and hemoglobin elevates IOP [27],[28]; and that transitory IOP elevation may result from breath holding and thorax compression while tonometry is performed in the sitting position [32].

Although BMI and IOP are known to be strongly associated, the data on the relationship between BMI and glaucomatous optic neuropathy are inconsistent [17],[32]. Supporting many of the previous reports, the results of the current study showed that significant IOP elevation did not result in glaucomatous optic neuropathy, which, despite the possibility that IR may be associated with slight IOP elevation, suggests that IR by itself is not a risk factor for glaucoma. Because no previous research has compared the association between IOP and IR in non-obese and obese subjects, further verification is required before any firm conclusions can be drawn.

This study faced several limitations and strengths that should be considered when reviewing the results. One limitation was the use of a cross-sectional design, which did not allow for identification of any possible causal relationships. Determination of causal relationships, if any, should thus be pursued by conducting additional prospective studies. Another limitation was that the data regarding lifestyle factors may have been affected by recall bias, as they were collected via self-reported questionnaires. A third limitation was that endogenous levels of cortisol and other steroidal hormones, which cause morphologic and biochemical changes in the trabecular meshwork and the ocular tissue involved in regulating IOP [33]; and of hormones related to MetS, such as adipokines, resistin, leptin, and inflammatory cytokines, were not evaluated in the study [34]. Lastly, although the role of bones as endocrine organs, and especially the role of osteocalcin in insulin resistance are now fully recognized [35],[36], we did not consider the bone mass of each patient enrolled in this study, nor the blood concentration of osteocalcin.

On the other hand, a major strength of this study was the examination of a nationally representative sample of the adult population of South Korea, which is subject to relatively uniform genetic and environmental influences, including climate and diet. Another strength was the use of a standardized protocol that called for the use of standardized procedures for conducting clinical assessments, anthropometric measurements, and biochemical analyses. A major finding of this study is the positive correlation between IOP and IR in non-obese but not in obese subjects, a finding that potentially has significant clinical and public health implications. One potential implication is that, despite being well-defined pathogenic factors in ocular hypertension, obesity and IR may contribute independently to IOP elevation. Although the IOP elevation observed in this study was not sufficient to diagnose ocular hypertension, and although mere IOP elevation is neither necessary nor enough for diagnosing glaucoma, it is noteworthy that our findings suggest that IR improvement by lifestyle intervention may show therapeutic potential in decreasing IOP, at least in non-obese individuals. This implication in turn suggests that improving the MetS syndrome component of IR might not be helpful in preventing IOP elevation without simultaneously pursuing weight loss and/or control. Further studies are required to clarify the causal relationship between IOP and IR according to obesity status. Lastly, as endocannabinoids may play a role in adipogenesis [37], detailed evaluation of the link between the endocannabinoid system and food intake, lipid metabolism, and IOP should be considered in future research.
